# Explicit vs. implicit spatial processing in arrow vs. eye-gaze spatial congruency effects

**DOI:** 10.1007/s00426-022-01659-x

**Published:** 2022-02-22

**Authors:** Cristina Narganes-Pineda, Ana B. Chica, Juan Lupiáñez, Andrea Marotta

**Affiliations:** Department of Experimental Psychology, Mind, Brain, and Behavior Research Center (CIMCYC), University of Granada, Campus de Cartuja, s/n, 18071 Granada, Spain

**Keywords:** Arrows, Gaze, Implicit processing, Attentional orienting, Social attention, Reversed congruency effect, Spatial interference task

## Abstract

Arrows and gaze stimuli lead to opposite spatial congruency effects. While standard congruency effects are observed for arrows (faster responses for congruent conditions), responses are faster when eye-gaze stimuli are presented on the opposite side of the gazed-at location (incongruent trials), leading to a reversed congruency effect (RCE). Here, we explored the effects of implicit vs. explicit processing of arrows and eye-gaze direction. Participants were required to identify the direction (explicit task) or the colour (implicit task) of left or right looking/pointing gaze or arrows, presented to either the left or right of the fixation point. When participants responded to the direction of stimuli, standard congruency effects for arrows and RCE for eye-gaze stimuli were observed. However, when participants responded to the colour of stimuli, no congruency effects were observed. These results suggest that it is necessary to explicitly pay attention to the direction of eye-gaze and arrows for the congruency effect to occur. The same pattern of data was observed when participants responded either manually or verbally, demonstrating that manual motor components are not responsible for the results observed. These findings are not consistent with some hypotheses previously proposed to explain the RCE observed with eye-gaze stimuli and, therefore, call for an alternative plausible hypothesis.

## Introduction

The ability of human beings to perceive, attend, and adequately respond to other people’s gaze direction has been crucial to their survival. This ability provides a valuable source of information about what others are attending to and may have evolved from the need to detect food, predators, and other sources of threat (Emery, 2000).

Thus, gaze does not only allows us to explore our environment and extract relevant information, but has also a critical non-verbal communicative function (Itier & Batty, 2009). Through gaze, we can communicate socially relevant information, such as our focus of interest, private thoughts, emotions, and intentions (e. g., Baron-Cohen et al., 1997). According to Baron-Cohen’s account for social cognition (1995), the human brain has developed a specialized module called “eye-direction detection”, which serves to identify the presence and direction of gaze, as well as eye-contact (Macrae et al., 2002).

During visual search tasks, direct gaze (which refers to situations in which the eye-gaze stimuli and the participant’s gaze make visual contact) is detected faster than averted gaze. Direct gaze or eye-contact facilitates judgments regarding different aspects of the eyes or faces. According to the literature, direct gaze (as compared to averted gaze) improves attentional orienting towards the faces (Mares et al., 2016), and improves the discrimination of emotions (Hamilton, 2016; Hietanen et al., 2008; McCrackin & Itier, 2019); in particular, angry and happy faces are better detected under conditions of direct gaze as compared to averted gaze, while fearful faces are detected more frequently under conditions of averted gaze as compared to direct gaze in an attentional blink task (Adams & Kleck, 2005; Milders et al., 2011).

Similarly, gender categorization of human faces is facilitated when gaze is directed toward the observer (i.e., a direct gaze), compared with situations where gaze is averted or the eyes are closed (Burra et al., 2018; Macrae et al., 2002). Furthermore, direct gaze facilitates social categorization of faces, according to either race or identity (Kloth et al., 2015; Macrae et al., 2002; Richeson et al., 2008). Direct gaze is also processed faster than averted gaze during unconscious processing (Chen & Yeh, 2012). All these observations suggest that the perception of direct eye-gaze triggers processes of preferential detection and a better allocation of attentional resources, which modulate cognitive processing and behavioural responses (which is known as the eye-contact effect, Conty et al., 2007; Senju & Johnson, 2009). Contrary, averted gaze causes an automatic shift of attention towards the observed direction. This ability to attend to the same object or place where another person is looking at is called “joint attention”, a crucial process for the typical development of many social skills such as language and the theory of mind (Conty et al., 2007; Hietanen et al., 2008).

In the last years, researchers have tried to evaluate the uniqueness of attentional mechanisms triggered by gaze cues, trying to dissociate attentional mechanisms triggered by gaze from those engaged by symbolic non-social directional stimuli such as arrows. It is important to note that arrow cues have a directional property, just like gaze, but no biological or social significance (Birmingham & Kingstone, 2009; Capozzi & Ristic, 2018). Most studies have used variants of the traditional spatial cueing paradigm (Chica et al., 2014; Posner, 1980) to compare attentional engagement produced by eye-gaze and arrows. In the spatial cueing paradigm, either eyes or arrows are used as attentional cues, presented at fixation, followed by targets presented at either the left or right location. Both gaze and arrow cues result in faster reaction times (RT) to targets appearing at the cued location compared to other locations (the so-called gaze/arrow cueing effect), even when eyes or arrows are not predictive of the subsequent target location, and even when the time interval between the presentation of the cue and the target is very short (for reviews see Birmingham & Kingstone, 2009; Capozzi & Ristic, 2018). This suggests the relatively reflexive nature of this effect. However, using the spatial cueing paradigm, subtle or no behavioural differences have been generally observed between eye-gaze and arrow cues, leading some authors (Heyes, 2014; Santiesteban et al., 2014) to propose that gaze attentional effects are at least partially driven by a domain-general attentional process.

However, a different pattern of results emerges in paradigms aiming at investigating qualitative differences between gaze and arrows. For example, whereas gaze direction orients attention to the specific spatial location or part of the object looked at, arrows spread attention through the whole cued object (Chacón-Candia et al., 2020; Marotta et al., 2012). Gaze seems to selectively focus attention on and automatically select the specific location/part of the object looked at, rather than simply initiating the orienting of attention, as arrows do (Marotta et al., 2013). Moreover, combining a traditional gaze cueing paradigm with a visual working memory task, eye-gaze but not arrow cues enhanced visual working memory accuracy for cued information (Dodd et al., 2012; Gregory & Jackson, 2017). Thus, arrows and gaze seem to orient attention similarly towards the indicated/looked at direction. However, gaze seems to go beyond attentional orienting and further trigger location/object selection and its transfer into working memory.

Another paradigm has recently been used to more clearly dissociate the attentional effects of arrows and eye-gaze. Using a variant of the spatial Stroop task, in which eye-gaze or arrows were used as target stimuli, Marotta et al. (2018) observed that arrow and eye-gaze stimuli led to opposite spatial interference effects. In this paradigm, participants had to discriminate the direction of the targets (arrows or eye-gaze) unpredictably appearing to the left or right of the fixation point. Consistent with a Spatial Stroop effect (Lupiáñez & Funes, 2005), arrows elicited faster responses when their direction was congruent with their position (e.g., a left-pointing arrow presented to the left; typical Spatial Stroop or spatial congruency effect), whereas eye-gaze stimuli produced faster reaction times (RTs) when they were incongruent (e.g., a left looking eye-gaze stimulus presented to the right; which we refer to as the reversed Spatial Stroop or Reversed Congruency Effect; RCE). As stated by the authors (Marotta et al., 2018), this dissociation is difficult to reconcile with the domain-general view of attentional processes and seems more coherent with the view that attention to gaze represents a unique attentional process that is qualitatively distinct from the attentional mechanisms engaged by biologically irrelevant stimuli. The congruency effect produced by arrows has been explained by the interference generated between the relevant spatial dimension of the target (the directionality of the arrow) and its irrelevant spatial dimension (the location in which the arrow is presented) (Kornblum et al., 1990; Luo & Proctor, 2013).

Although several explanations have been proposed to explain the RCE observed with eye-gaze stimuli, the eye-contact hypothesis is one of the most plausible hypotheses (Cañadas & Lupiáñez, 2012; Marotta et al., 2018, 2019). This hypothesis states that when, for example, a gaze stimulus is presented to the left, looking to the right (incongruent trial), it is looking towards the centre, at the location the participant is looking at, therefore potentially making visual contact with the participant. On the contrary, if the face appears on the left, looking to the left (congruent trial), it is looking away from the participant (averted gaze). According to Cañadas and Lupiáñez (2012) and Marotta et al. (2018), the eye-contact effect could explain the RCE, with faster RTs when the gaze is looking at you (incongruent trial) as compared to the situation of averted gaze (congruent trial). This social effect is not present when arrows are used as targets. Although this hypothesis has been used to explain these results in previous studies, the eye-contact hypothesis has not been directly tested (Cañadas & Lupiáñez, 2012; Marotta et al., 2018). Alternatively, the RCE could also be explained by the motivational tendency to approach and/or to establish a social interaction during incongruent trials (direct gaze), while there is a motivational tendency to avoid the observer for congruent trials (averted gaze) (Hietanen et al., 2008). Another possible explanation suggests that on incongruent trials, the gaze is looking at the fixation point, at the same time that the participant is also orienting his/her gaze to the fixation point so that the participant and stimulus share the same object of attention, which would cause “joint attention”, facilitating the processing of the gaze on incongruent trials (Cañadas & Lupiáñez, 2012; Edwards et al., 2020). Furthermore, given that responses are also lateralized in this paradigm, the effect could also be due to some facilitation occurring in incongruent gaze trials, due to the incongruent gaze being perceived as looking at the correct response at the opposite side.

Moreover, it is well-known that social stimuli are often processed automatically and implicitly without much conscious effort (Lieberman, 2007), and the perception of gaze triggers automatic attentional orienting, even when the observer has neither the motivation nor the intention to direct his/her attention in that direction (Driver et al., 1999; see also Sato et al., 2007, 2016; Stein et al., 2011; Xu et al., 2011; Yokoyama et al., 2014). The eye-contact effect can occur implicitly, without much conscious effort, even when we do not intend to process direct gaze (Sato et al., 2007, 2016; Stein et al., 2011; Xu et al., 2018). However, to date, eye-gaze direction has been used as a task-relevant dimension in all the studies investigating the spatial interference paradigm with faces and eyes (Cañadas & Lupiáñez, 2012; Jones, 2015; Marotta et al., 2018; Torres-Marín et al., 2017), and the effect of the implicit or incidental processing of eye-gaze direction on the RCE has not been explored yet. It is still unknown whether and how eye-gaze direction can affect behaviour even when it is not task-relevant in spatial interference tasks. Therefore, in the present study, we intendedly used an implicit task in which direction was not task-relevant. If the eye-contact effect were responsible for the RCE observed with eye-gaze stimuli, this effect should be observed both in the explicit task (where the task-relevant dimension was the direction of the stimuli, eye-gaze or arrow) but also in the implicit task (where the relevant dimension was colour, while direction was completely irrelevant for the task). Supporting this idea, there is evidence showing that eye-contact accelerates responses in a colour-discrimination task (Hietanen et al., 2016), and it is observed even in a detection task (Song et al., 2021; with lateralized faces or eyes), while gaze was completely irrelevant to the task (and participants did not even have to look at the faces or eyes). On the other hand, if no RCE were observed in the implicit task, the RCE could be entirely explained by the eye-contact hypothesis because eye contact should occur in this task, no matter whether the direction is or is not task-relevant. This is also theoretically important because the observation of a RCE only with explicit gaze processing would indicate the implication of mechanisms other than those involved in gaze cueing, which are observed with both implicit and explicit gaze processing (Sato et al., 2007).

Therefore, the aim of this study was to investigate the effect of implicit processing of eye-gaze direction on spatial interference effects in general and on the RCE in particular. This study is essential to elucidate the nature of the reverse congruency effect observed with eyes, and to further understand the boundary conditions in which eye-gaze direction can affect behaviour. In three experiments, we employed both explicit and implicit spatial interference tasks to test our hypothesis: if an eye-contact effect underlies the RCE with eye-gaze stimuli, this effect should be observed both in the explicit and implicit version of the task with eye-gaze stimuli, since eye contact should always occur, even when participants do not pay attention to stimuli direction or the processing of direction is incidental or implicit (Rothkirch et al., 2015; Sato et al., 2007; Stein et al., 2011; Xu et al., 2018; Yokoyama et al., 2014). For arrow stimuli, a typical congruency effect is expected in the explicit task, while no congruency effect is expected in the implicit task. In the latter, no interference is expected as the relevant dimension (colour) does not overlap with the two irrelevant spatial dimensions (location and directionality) (Kronblum, 1990; Luo & Proctor, 2013).

## Experiment 1

The goal of Experiment 1 was to replicate previous studies showing opposite congruency effects with eye-gaze and arrow stimuli (Cañadas & Lupiáñez, 2012; Marotta et al., 2018) and to test whether the reversed congruency effect is still observed with eye-gaze stimuli when both space and direction are implicitly processed. To this aim, participants were required to identify the direction (explicit task), or the colour (implicit task) of both eyes-gaze and arrows, which were presented to either the left or right of the fixation point. Furthermore, the experiment was run as a pilot for a functional Magnetic Resonance Imaging (fMRI) study. Therefore, some parameters were changed from the original studies to adapt the procedure to fMRI.

### Method

#### Participants

A total of 48 participants (4 men, mean age = 20.44, SD = 2.67) from the Faculty of Psychology, University of Granada, participated voluntarily in the present experiment in exchange for course credit.

In Experiment 1, and the subsequent experiments, we estimated sample sizes based on previous research with the spatial interference paradigm. Marotta et al.’s (2018) study, which had enough statistical power to observe the critical effects (the interaction between Target Type and Congruency, and for each critical planned comparison), used a sample size of 36 participants. However, a posteriori sensitivity power analysis using G^∗^power (Faul et al., 2007), showed that with a sample size of *n* = 48, the minimum effect size that could be detected for *α* = 0.5, and 1 − *β* = 0.95, for 2 groups and 4 within-participants conditions (for the critical Target Type × Congruency interaction) was *η*^*2*^*p* = 0.04 (minimum detectable effect). Therefore, 24 participants per group were sufficient to observe the critical interaction (Target Type × Congruency), which effect size was larger than *η*^*2*^*p* = 0.09 in both Experiments 1 and 3.

All participants were naive about the purpose of the experiment and reported having normal or corrected to normal vision. Signed informed consent was collected before the study, and participants were informed about their right to withdraw from the experiment at any time. The ethics committee from the University of Granada approved the experiments (175/CEIH/2017). Half of the participants (*n* = 24) were randomly assigned to the explicit task, while the other half (*n* = 24) were assigned to the implicit task.

#### Apparatus and stimuli

**Fig. 1:**
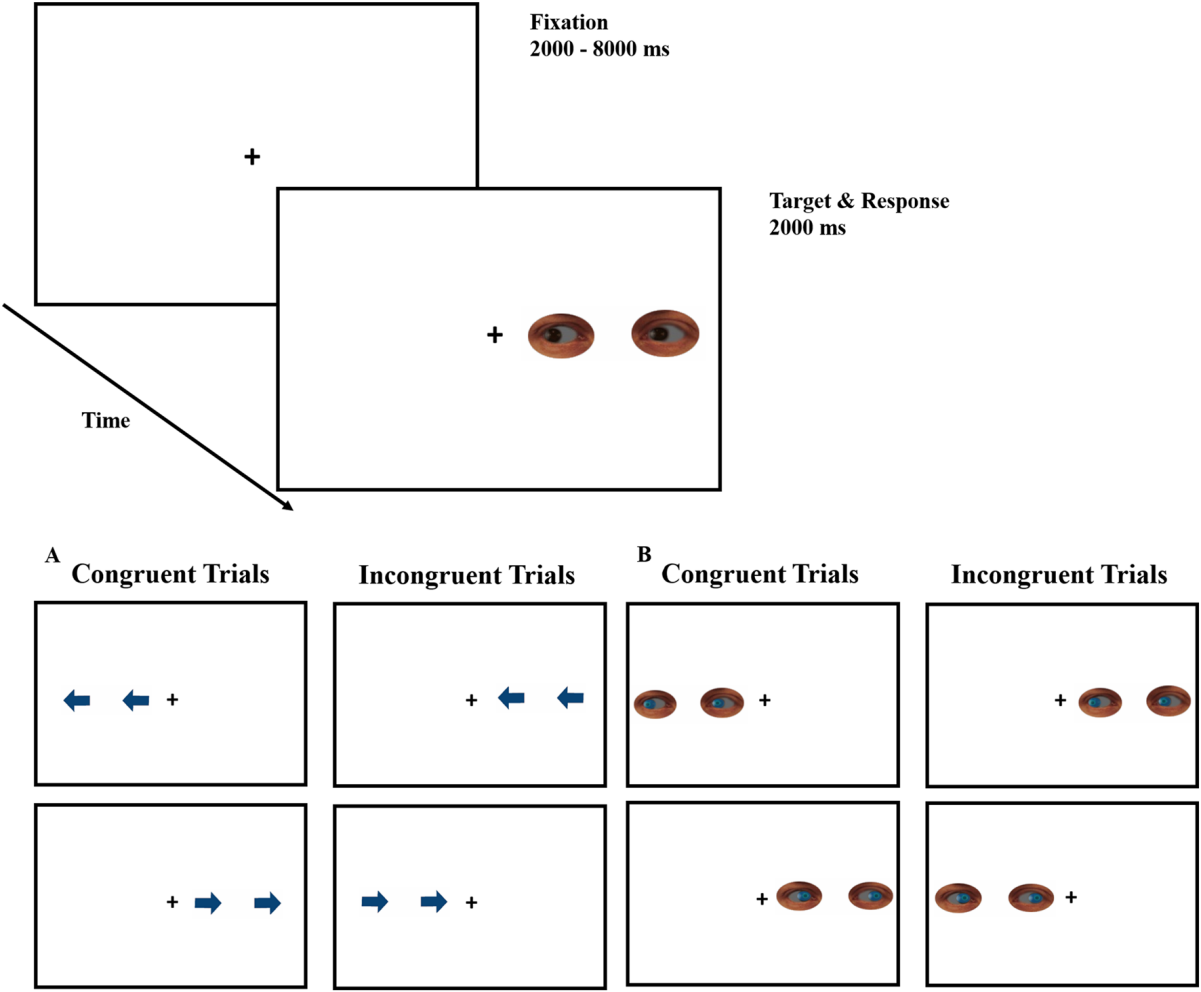
Schematic representation of the trial sequence of Experiment 1. In the example, an incongruent eye-gaze trial is represented. Bottom part: representation of all possible trials (congruent and incongruent with blue stimuli are represented, but stimuli could also be brown in colour) for **A** arrows, and **B** eye-gaze stimuli. The size of the eye-gaze stimuli has been increased to facilitate visibility, i.e. stimuli are not represented at scale

Stimulus presentation, timing, and data collection were controlled using E-Prime 2.0 (Schneider et al., 2002), ran on a standard Pentium 4 PC. Stimuli were presented on a 17″ widescreen monitor with a 1024 × 768-pixel resolution. All stimuli were presented on a white background. In each trial, a fixation point was presented at the centre of the screen. The target consisted of a 1.5 × 6.5 cm display of two arrows or two full cropped eyes, presented to either the left or right of the fixation point, and which could be either blue or brown (see Fig. 1). The distance from the fixation point to the centre of the lateral stimulus was 7 cm. Stimuli could be either blue or brown. Cropped eyes were obtained by manipulating the original stimuli from the NimStim Set of Facial Expressions (https://danlab.psychology.columbia.edu/content/nimstim-set-facial-expressions) with Adobe Photoshop CS.

#### Procedure

Before starting the experiment, all participants provided their written informed consent to participate in the study. They sat at an approximate distance of 57 cm from the computer screen in a dark room.

The experiment consisted of two blocks of trials (one for each target type), each one composed of 15 practice trials followed by two experimental blocks of 64 trials each. The order of the two blocks was counterbalanced across participants. Since the experiment was designed for being a pilot fMRI study, the jitter fixation and the order of trial types were determined with an optimal sequencing programme designed to maximize the efficiency of recovery of the Blood-Oxygen-Level Dependent (BOLD) response (Optseq II). The jitter fixation periods were interleaved with the experimental trials as determined by the optimization programme.

Each trial started with a black fixation point for a random interval ranging from 2000–8000 ms (in 2000 ms steps). Participants were instructed to keep their eyes on the fixation point throughout the experiment. The target was then presented (two arrows or two eyes), for 2000 ms, pointing either to the right or to the left, and appearing either to the left or the right location.

In the explicit task, participants responded to the direction (left or right) the arrow or the eye-gaze stimulus was pointing at (by pressing either the “*Z*”–left, or the “*M*”–right, key on the QWERTY keyboard). In the implicit task, participants were asked to perform a colour-discrimination task by pressing one of the keys (“*Z*” or “*M*”) for blue stimuli and the other one for brown stimuli, depending on the counterbalanced condition. In both conditions, participants were instructed to respond as fast and accurately as possible within the 2000 ms of stimulus onset.

#### Design

A three-factor mixed design was used to analyse the data. Target type (arrow vs. eye-gaze) and Congruency (congruent vs. incongruent trials) were manipulated within participants, while Task (explicit vs. implicit) was manipulated between participants. For each participant, a total of 64 observations per experimental condition were considered. Practice trials were not analysed. Mean RTs and accuracy were calculated for each experimental condition and were used as dependent variables. Planned comparisons were used for the analysis of interactions.

### Results

Trials with an incorrect response (3.22%), correct response trials with RTs faster than 200 ms (0%) or slower than 1300 ms (1.38%) (Marotta et al., 2018), were considered anticipations and lapses, respectively, and were excluded from the RT analysis. Mean RTs and percentages of errors for each experimental condition are shown in Table 1.

**Table 1:** Mean correct RTs (in ms) and percentage of incorrect responses (IR) (with their corresponding standard deviations, SD), for each experimental condition of Experiment 1

		Arrow				Eye-Gaze			
		RT	SD	%IR	SD	RT	SD	%IR	SD
Explicit Task	Congruent	504	85	0.80	1.07	598	87	2.53	4.67
	Incongruent	535	76	3.84	4.22	584	81	3.70	3.25
Implicit Task	Congruent	615	100	2.75	2.25	642	97	2.40	2.17
	Incongruent	611	102	3.11	2.54	638	92	2.68	2.12

Mean RT data were submitted to a 2 (Task) × 2 (Target Type) × 2 (Congruency) mixed ANOVA, with Task (explicit vs. implicit) as a between-participant factor, and Target Type and Congruency as within-participants’ factors. The analysis revealed a main effect of Target Type, *F*(1,46) = 93.32, MSE = 1248, *p* < 0.001, *η*^*2*^*p* = 0.67, and a main effect of Task, *F*(1,46) = 7.92, MSE = 30,785, *p* = 0.007, *η*^*2*^*p* = 0.15. Shorter RTs were observed for arrows (566 ms) than eye gaze targets (616 ms), and for the explicit task (555 ms) than the implicit task (627 ms). The main effect of Congruency was not significant, *F* < 1, but, critically, the interaction between Target Type and Congruency was significant, *F*(1,46) = 14.55, MSE = 388, *p* < 0.001, *η*^*2*^*p* = 0.24. The Target Type × Task and Congruency × Task interactions were also significant (respectively, *F*(1,46) = 18.88, MSE = 1248, *p* < 0.001, *η*^*2*^*p* = 0.29 and *F*(1,46) = 5.53, MSE = 355, *p* = 0.023, *η*^*2*^*p* = 0.11), showing larger effects of Target Type and Congruency for the explicit task than implicit task.

**Fig. 2:**
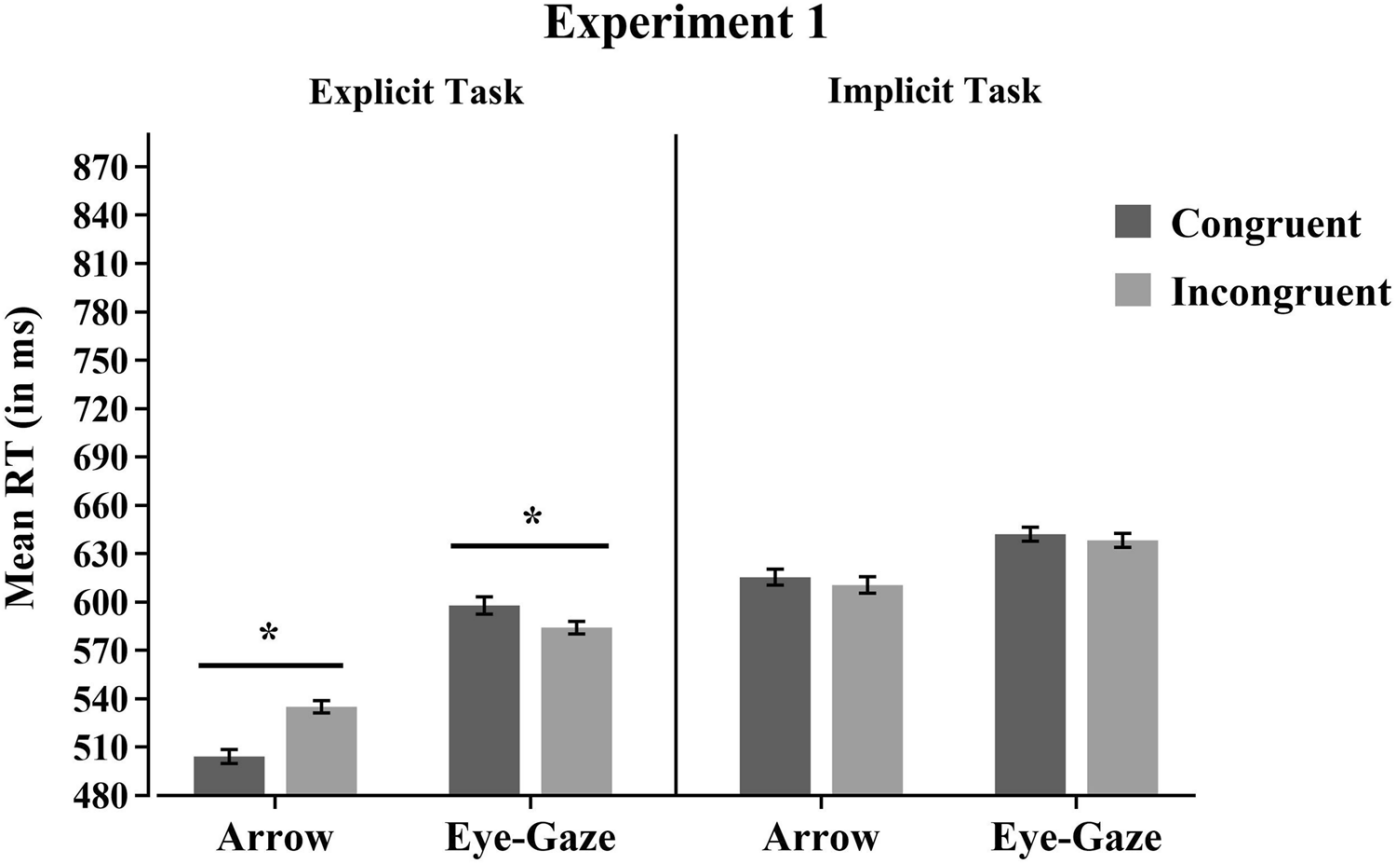
Mean RT for each Target Type, Congruency, and Task condition of Experiment 1. Asterisks represent statistically significant planned comparisons. Cousineau’s method (2005) was used to calculate the standard errors of the means represented as error bars

The critical three-way interaction between Target Type, Congruency, and Task was significant, *F*(1,46) = 16.12, MSE = 388, *p* < 0.001, *η*^*2*^*p* = 0.26, converging on the conclusion that the Interaction between Target Type and Congruency was modulated by the type of task. To explore this interaction further, separate ANOVAs were conducted for each task. The ANOVA for the Explicit Task revealed a significant Target Type × Congruency interaction, *F*(1,23) = 34.08, MSE = 349, *p* < 0.001, *η*^*2*^*p* = 0.60. Planned comparisons showed that with arrow stimuli, RTs were significantly slower for incongruent (535 ms) than for congruent trials (504 ms) *F*(1,23) = 46.42, MSE = 244, *p* < 0.001, *η*^*2*^*p* = 0.67. In contrast, for eye-gaze stimuli, RTs were significantly faster for incongruent (584 ms) than for congruent trials (598 ms), *F*(1,23) = 4.43, MSE = 513, *p* = 0.046, *η*^*2*^*p* = 0.16 (the so-called RCE). In contrast, in the Implicit Task, the interaction between Target Type and Congruency was not significant *F* < 1. Planned comparisons revealed no significant differences between congruent and incongruent trials neither with arrow stimuli, *F* < 1, nor with eye-gaze stimuli, *F* < 1 (Fig. 2).

The analysis of errors showed a main effect of Congruency, *F*(1,46) = 11.54, MSE = 6 *p* = 0.001 *η*^*2*^*p* = 0.20, with more errors for incongruent (3.33%) than congruent trials (2.12%). No other main effect was significant (all Fs < 1). The only significant interaction was Congruency × Task, *F*(1,46) = 6.28, MSE = 6, *p* = 0.016 *η*^*2*^*p* = 0.12. Neither the interaction between Target Type and Congruency (*F*(1,46) = 2.51, MSE = 5 *p* = 0.12, *η*^*2*^*p* = 0.05), nor the Target Type × Congruency × Task interaction (*F*(1,46) = 2.13, MSE = 5, *p* = 0.15, *η*^*2*^*p* = 0.04) were significant.

### Discussion

In this experiment, we explored whether the RCE observed with eye-gaze stimuli was also found even when the direction of stimuli was not relevant for the task. In the explicit task, in which participants were required to respond to the direction of stimuli (eye-gaze and arrows), we replicated the congruency effect with arrows and the RCE with eye-gaze stimuli (Marotta et al., 2018). Whereas arrows elicited the typical spatial interference effect (i.e., faster RTs when their position was congruent with the direction the arrow was pointing at), eye-gaze produced a RCE (i.e., faster responses for incongruent as compared to congruent trials). According to Cañadas and Lupiáñez (2012) and Marotta and et al., (2018), the effect observed with eye-gaze stimuli in RTs can be explained by faster responses when eye contact is maintained. Note that when eye-gaze trials were incongruent (i.e., when eye-gaze was presented on the right looking to the left), stimuli were looking at the centre, putatively favouring eye-contact with participants.

Moreover, as in Marotta et al., (2018), slower RT was observed with eye-gaze stimuli compared to arrows. This was probably due to the fact that eye-gaze stimuli are more difficult to process due to their social significance and/or perceptual complexity (Hietanen et al., 2006).

Importantly, no congruency effects were observed with either arrow or eye-gaze stimuli when a colour-discrimination task was required (implicit task). This finding suggests that, unlike what happens with gaze cueing paradigms (Sato et al., 2007), in the spatial interference paradigm, eye-gaze direction is not able to affect behaviour when it is implicitly processed. Therefore, these findings are not consistent with the eye-contact hypothesis since eye contact was not prevented in the implicit task, and nevertheless no RCE was observed. If the eye-contact occurred on incongruent trials, causing the RCE in the explicit task, it should have also occurred in the implicit task, because eye-contact also happened there.

Furthermore, although several studies have observed that eye-contact produces an increased attentional orienting, as well as more eye movements than averted gaze, even when the gaze is implicitly or subliminally processed (Chen & Yeh, 2012; Madipakkam et al., 2015; Stein et al., 2011), there is less evidence about whether and how eye-contact implicitly modulates social cognition. Some studies suggested different eye-contact functions with and without awareness in social decision-making (Luo et al., 2016) and on gaze cueing effects (Xu et al., 2018). A similar dissociation could also apply to the present study, explaining the different results observed between the explicit and the implicit conditions. However, further studies are necessary to shed light on this issue. Moreover, since no previous studies have explored the congruency effects with an implicit task, a replication seemed mandatory.

## Experiment 2

In the previous experiment, no congruency effects were observed when participants were required to discriminate the colour (implicit task) instead of the direction of the target (explicit task). However, Experiment 1 was, to the best of our knowledge, the first experiment that has ever assessed congruency effects elicited by eye-gaze and arrow stimuli by means of an implicit version of the spatial interference task. Therefore, it seems essential to replicate it with a different and larger sample. This was the primary purpose of Experiment 2. Moreover, it is important to note that in our previous experiment, the procedure, timing, and stimuli were adapted for piloting a future fMRI study, which could have influenced the observed pattern of results. For this reason, in Experiment 2, while some participants performed the implicit task with the same stimuli and procedure of Experiment 1, others performed the implicit task with a procedure and timing similar to the ones used in Marotta et al.’s (2018) study (see “Method” section for more details).

### Method

#### Participants

A total sample of 76 participants (11 males, mean age = 20.20, SD = 2.36) from the Faculty of Psychology of the University of Granada participated voluntarily in the experiment in exchange for course credit. In Experiment 2a, twenty-four participants were tested using the same stimuli, timing, and procedure of the implicit task of Experiment 1 (using an identical sample size). In Experiment 2b, fifty-two participants were tested using a design with timing and procedure similar to Marotta et al.’s (2018). Given that a null result was expected in the implicit task, we increased sample size to acquire evidence in favour of the null or alternative hypothesis using Bayesian statistics. A sensitivity power analysis using *G* ∗ power (Faul et al., 2007) showed that with our final sample size (*n* = 76), the minimum effect size that could be detected for *α* = 0.5, and 1 − *β* = 0.95, for two groups and four within-participants conditions was *η*^*2*^*p* = 0.028 (minimum detectable effect).

#### Apparatus and stimuli

In Experiment 2a, apparatus and stimuli were identical to those used in the implicit task of Experiment 1. In Experiment 2b, apparatus and stimuli were the same, but stimuli were presented at an eccentricity of 8 cm (instead of 7 cm) from the fixation point.

#### Procedure

In Experiment 2a, the procedure and timing were identical to those of the implicit task of Experiment 1. In Experiment 2b, procedure and timing were adapted from Marotta et al.’s (2018) study. Practice trials had feedback for incorrect keypresses in the form of a 220 Hz tone. Each trial started with a black fixation point presented in the centre of the screen for 1000 ms. The target (either two arrows or two eyes) was then presented for a fixed period of 1500 ms. As in the implicit task of Experiment 1 and Experiment 2a, target stimuli could be blue or brown randomly. Participants were asked to perform a colour-discrimination task by pressing as fast and accurately as possible one of the keys (“*Z*” or “*M*”) for blue stimuli and the other one for brown stimuli, depending on the counterbalance condition.

#### Design

In a preliminary analysis, the design consisted of a three-factor mixed measures design with the following factors: Experiment (2a and 2b), Target type (arrow and eye-gaze), and Congruency (congruent and incongruent). However, since the main effect of Experiment or its interaction with other variables was not significant (all ps > 0.09, with the three-way Target Type × Congruency × Experiment interaction far from significance, *F* < 1), this factor was eliminated from the remaining analyses. Therefore, we used the same design of the implicit condition of Experiment 1.

### Results

As in Marotta et al., (2018), trials with RTs faster than 200 ms (0.01%) or slower than 1300 ms (0.99%) as well as incorrect responses (3.74%) were excluded from the RT analysis. Table 2 shows the mean (and standard deviation) for RTs and percentages of errors for each experimental condition.

**Table 2:** Mean correct RTs (in ms) and percentage of incorrect responses (IR) (with the corresponding standard deviations SD) for each experimental condition of Experiment 2

		Arrow				Eye-Gaze			
		RT	SD	%IR	SD	RT	SD	%IR	SD
Implicit Task	Congruent	600	85	3.34	3.72	636	73	2.97	3.35
	Incongruent	603	80	3.38	3.16	634	76	2.94	2.85

**Fig. 3:**
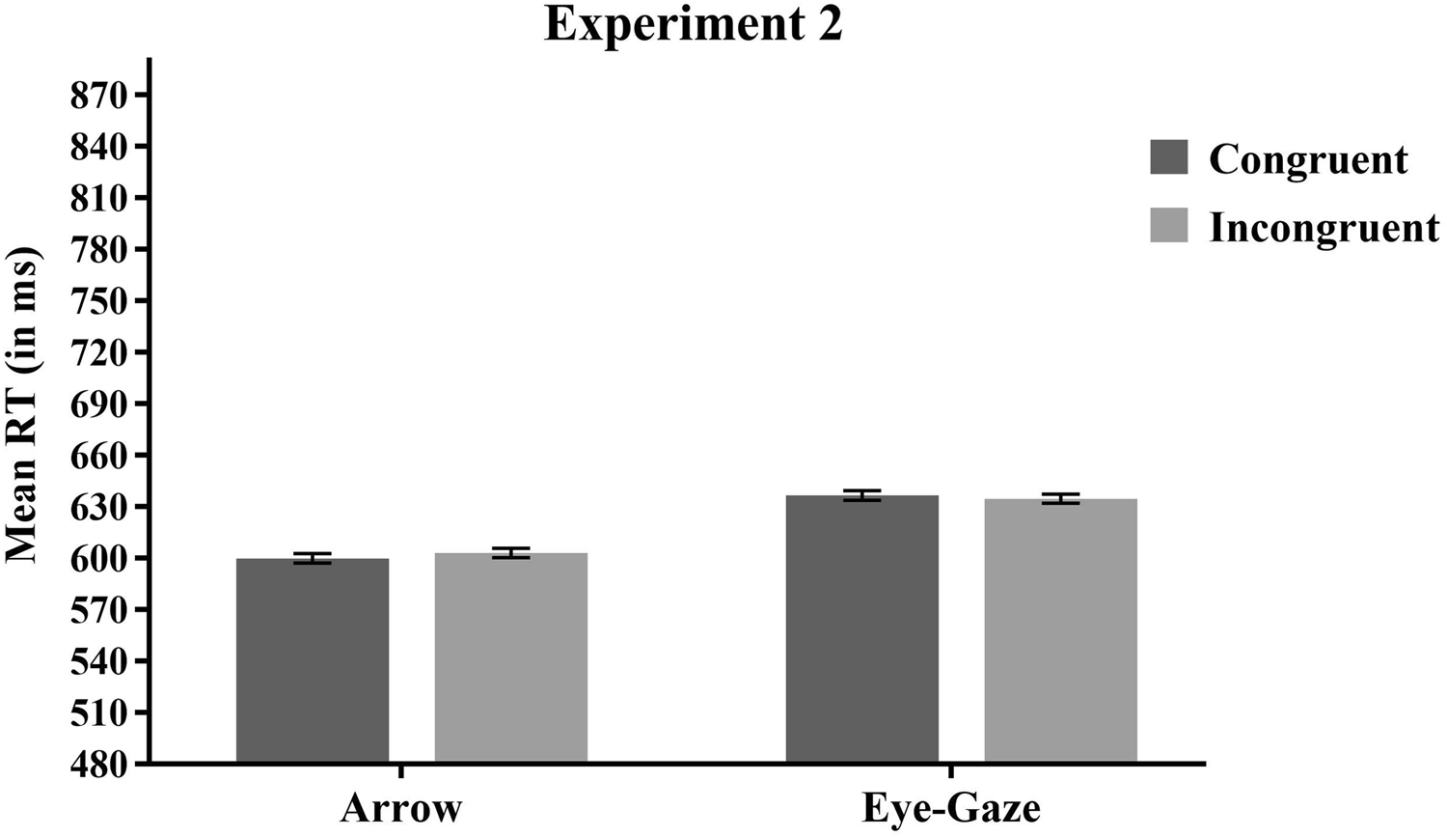
Mean RT for each Target Type and Congruency condition of Experiment 2. Cousineau’s method (2005) was used to calculate the standard errors of the means represented as error bars

Mean RT data were submitted to 2 (Target Type) × 2 (Congruency) repeated measure ANOVA. The analysis of the mean RTs again revealed a main effect of Target Type *F*(1,75) = 52.15, MSE = 1698, *p* < 0.001, *η*^*2*^*p* = 0.41, with shorter RTs for arrow targets (601 ms) than for eye-gaze targets (635 ms). The main effect of Congruency, and the Target Type × Congruency interaction were not significant, *F* < 1 and *F*(1,75) = 1.52, MSE = 326, *p* = 0.222, *η*^*2*^*p* = 0.02, respectively. Planned comparisons confirmed that there were no significant differences between congruent and incongruent trials neither with arrow stimuli, *F*(1,75) = 1.06, MSE = 348, *p* = 0.304, *η*^*2*^*p* = 0.01, nor with eye-gaze stimuli, *F* < 1 (see Fig. 3).

In the analysis of errors, neither the main effects nor the interaction were significant (all *ps* > 0.17).

#### Combined analysis of experiments 1 and 2

This section presents a combined analysis of the implicit task of Experiment 1 and Experiment 2 (*N* = 100) to check and confirm the absence of congruency effects for the implicit task.

Mean correct RTs were submitted to a two-factor repeated measures design with Target Type (arrows and eye-gaze) and Congruency (congruent and incongruent) as within-participants’ factors. As reported above, Experiment did not modulate participants’ performance, and therefore, it was not included as a factor in the combined analyses (all ps > 0.12).

Moreover, the combined analysis was also performed with Bayesian statistics to check for evidence in favour of either the null hypothesis (evidence for no main effect of congruency or no interaction between Target Type and Congruency). In Bayesian statistics, analyses are not biased against the null hypothesis. They allow quantifying how much the gathered evidence (i.e., the observed data) supports either the presence or the absence of an effect. Therefore, on the basis of Bayesian statistics, we can conclude whether the alternative hypothesis is more probable than the null hypothesis or vice versa. Following Wagenmakers et al. (2018), a BF_10_ = 1 indicates no evidence favouring the alternative hypothesis (H1) or the null hypothesis (H0). BF_10_ > 1 indicate evidence in favour of H1: 1–3 anecdotal evidence, 3–10 moderate evidence, 10–30 strong evidence. BF_10_ < 1 indicate evidence in favour of H0: 0.33–1 anecdotal evidence, 0.10–0.33 moderate evidence, 0.03–0.10 strong evidence. Output effects for the main effects and interactions in the Bayesian ANOVAs are presented across matched models, following Wagenmakers et al. (2018) recommendations for Bayesian analyses in JASP.

The analysis showed one more time a main effect of Target Type, *F*(1,99) = 65.15, MSE = 1615, *p* < 0.001, *η*^*2*^*p* = 0.40, *BF*_*inclusion*_ = 3.03852e + 23. The main effect of Congruency and the interaction between Target Type and Congruency were not significant (both Fs < 1; *BF*_*inclusion*_ = 0.11 and *BF*_*inclusion*_ = 0.19, respectively). The analysis of the interaction revealed no significant differences between congruent and incongruent trials neither with arrows, *F* < 1, *BF*_*10*_ = 0.16, nor with eye-gaze stimuli, *F*(1,99) = 1.13, MSE = 255, *p* = 0.29, *η*^*2*^*p* = 0.01, *BF*_*10*_ = 0.25. These analyses demonstrate that there is moderate evidence in favour of an absence of congruency effects for both arrow and eye gaze stimuli for the implicit task.

We further explored whether the main effect of Congruency, or the Congruency × Target Type interaction were observed in the implicit task while equating response conditions between the implicit and the explicit task. Note that while in the explicit task (in Experiment 1 and in Marotta et al., 2018) there is always a compatible mapping between the target direction and the response location (e.g., an eye-gaze/arrow indicating left always required a left response), in the implicit task participants were required to respond to the target colour independently from its direction and consequently keypresses responses could be consistent or not with target direction, i.e., a compatible mapping response was only required for the 50% of trials. As such, it is unclear whether the lack of Target Type × Congruency interaction observed in the implicit task condition was due to the implicit nature of the task or to the between tasks differences in response mapping. Indeed, it is possible that congruency effects are only present when the response mapping is compatible. To test for this possibility, we performed an ANOVA considering the response mapping as a factor in the combined analysis with 100 participants.

The analysis showed one more time a main effect of Target Type, *F*(1,99) = 65.15, MSE = 3231, *p* < 0.001, *η*^*2*^*p* = 0.40. The main effect of Congruency was not significant, *F* < 1. However, the main effect of Response Mapping was significant, *F*(1,99) = 10.56, MSE = 701, *p* = 0.002, *η*^*2*^*p* = 0.10, with faster responses for compatible (617 ms) than incompatible mapping response trials (623 ms). Neither the interaction between Target Type × Congruency, nor Target Type × Response Mapping were significant, both Fs < 1. Furthermore, and importantly for our research aims, the critical three-way Target Type × Congruency × Response Mapping interaction was not significant, *F*(1,99) = 1.07, MSE = 630, *p* = 0.30, *η*^*2*^*p* = 0.01.

Interestingly, the Congruency x Response Mapping interaction was significant, *F*(1,99) = 4.05, MSE = 1223, *p* = 0.047, *η*^*2*^*p* = 0.04, revealing no significant differences between congruent (615 ms) and incongruent trials (619 ms) on compatible response mapping trials, *F*(1,99) = 1.63, MSE = 587, *p* = 0.20, *η*^*2*^*p* = 0.02. In contrast, for incompatible response mapping trials RTs were significantly faster for incongruent (621 ms) than for congruent trials (626 ms), *F*(1,99) = 4,85, MSE = 319, *p* = 0.03, *η*^*2*^*p* = 0.05. This interaction can be easily explained by the fact that, while in the compatible mapping congruent trials are responded with the ipsilateral response and incongruent trials with the contralateral one, the opposite is true for the incompatible response mapping. So, this interaction, refer to a main Simon effect.

Indeed, when Simon laterality was considered in the analysis instead of Response Mapping, a main effect of Simon laterality was observed, *F*(1,99) = 4.05, MSE = 1223, *p* = 0.047, *η*^*2*^*p* = 0.04, with faster responses for ipsilateral (618 ms) than contralateral trials (623 ms). Again, and importantly, neither the Target Type × Congruency, nor the Target Type × Simon interaction or the three-way interaction were significant, all Fs ≤ 1. The Congruency x Simon interaction was significant, *F*(1,99) = 10.56, MSE = 701, *p* = 0.002, *η*^*2*^*p* = 0.1, revealing significant differences between congruent (615 ms) and incongruent trials (621 ms) in ipsilateral trials, *F*(1,99) = 4.14, MSE = 364, *p* = 0.04, *η*^*2*^*p* = 0.04. For contralateral trials the opposite was true, RTs were significantly faster for incongruent (619 ms) than for congruent trials (626 ms), *F*(1,99) = 7.92, MSE = 281, *p* = 0.006, *η*^*2*^*p* = 0.07. Again, this interaction represents the main effect of Response Mapping. Importantly, these analyses show that, although significant effects of Response Mapping and Simon laterality are observed when correspondingly analysed, they do not modulate neither the main effect of Congruency nor the critical Congruency × Target Type interaction.

Moreover, it is known that the response compatibility of the preceding trial affects the response mapping effect of the subsequent trial, with stronger effects following a preceding compatible than following a preceding incompatible trial. Therefore, a fairer test for the implicit task should restrict the analyses to the compatible response mapping trials, also preceded by another compatible response mapping trial, as this would mimic the conditions in the explicit task even better. To explore this issue, we reanalyzed the data filtering only trials in which a compatible response mapping was used in the current and the previous trial. Results revealed a non-significant, although marginal, Target Type × Congruency interaction, *F*(1,99) = 3.11, MSE = 1487, *p* = 0.08, *η*^*2*^*p* = 0.03. To further explore this issue, although being aware that the number of trials per condition is much reduced (average of 7.54 trials per experimental condition, ranging from 2 to 19), we reanalyzed the data only considering the trials with compatible response mapping on both the two previous trials and the current trial. Results now revealed a significant interaction between Target Type and Congruency, *F*(1,99) = 4.15, MSE = 2328, *p* = 0.044, *η*^*2*^*p* = 0.04, although the analysis of the interaction revealed no significant differences between congruent and incongruent trials neither with arrows (congruent: 588 ms, incongruent: 595 ms) nor with eye-gaze stimuli (congruent: 643 ms, incongruent: 630 ms).

### Discussion

Experiment 2 confirmed the absence of congruency effects in the implicit task when the direction of both arrow and eye-gaze stimuli was irrelevant for the task. This was true even when the same parameters of the original task (Marotta et al., 2018) were used. Indeed, the congruency effect was not modulated by the timing manipulation, size of the stimuli, or eccentricity. The absence of congruency effects in the implicit task suggests that the direction of both arrows and eye-gaze stimuli do not affect behaviour when it is implicitly processed in the spatial interference paradigm.

Nevertheless, an alternative explanation for the absence of congruency effects in the implicit task with both targets (arrow and eye-gaze) may be related to the task differences in response mapping. It is important to note that while in the explicit task there was always a compatible mapping between the target direction and the response location (e.g., an eye-gaze/arrow indicating left always required a left response), in the implicit tasks a compatible response mapping was only required in 50% of the trials. To explore this issue, we reanalyzed the data, considering the response mapping as a factor in the combined analysis. Although results demonstrated an effect of response mapping, with faster responses for compatible than incompatible response mapping trials, this effect did not modulate the crucial interaction between Target Type and Congruency, demonstrating that this effect was not different between eye-gaze and arrow stimuli. Therefore, the response mapping variable does not appears to be critical for the difference observed in the congruency effect between the two stimuli. Moreover, when we restricted the analysis to compatible response mapping preceded by compatible response mapping trials to mimic in the implicit task the scenario of the explicit task, the data are along the same lines; there is no interaction between Congruency and Target type. Only if we accumulate several compatible mapping response trials in a row do we find such a significant interaction (Target type × Congruency), which could be related to the task set implicitly induced by the repeated compatible response mapping. However, this is highly speculative and the analysis were not completely reliable, as there were not enough trials to properly explore this issue.

Similarly, when laterality of responses was instead considered in the analyses, a significant Simon effect was observed in the implicit task, with faster responses when stimulus location and response location matched, although again the effect was not modulated by Target type, which might contradict previous studies finding a “gaze direction Simon effect” (Zorzi et al., 2003). In any case, it is difficult to conclude whether or not the Simon or Response Mapping effects are implicated in the results observed with the implicit task, regarding the lack of a congruency effect.

Therefore, to more directly rule out the possibility that motor components are responsible for the difference in congruency effects observed between the explicit and implicit tasks, in Experiment 3, rather than controlling for, we decided to eliminate laterality of responses in both tasks. Thus, verbal (instead of manual) responses were required for both the implicit and explicit spatial interference tasks.

## Experiment 3

Experiment 3 was conducted to control for the possibility that manual motor components were responsible for the differences in the congruency effect observed between explicit and implicit tasks and to replicate the main findings obtained in the previous experiments. This experiment replicated Experiment 1, except that participants were required to respond verbally instead of manually. In particular, Experiment 3A consisted of a verbal explicit task, in which participants verbally reported the direction of the stimuli, and Experiment 3B consisted of a verbal implicit task, in which participants verbally reported the colour of the stimuli.

### Method

#### Participants

A new sample of 50 participants (13 males, mean age = 21.62, SD = 3.53) from the Faculty of Psychology of the University of Granada participated voluntarily in the experiments in exchange for course credit. Twenty-five participants took part in Experiment 3A, and twenty-five different participants in Experiment 3B.

#### Apparatus and stimuli

Apparatus and stimuli were identical to those used in Experiment 1, except for the use of a soundbox for response collection (RTs). The soundbox collected RT, using an ATR 20 microphone with low impedance connected to a Serial Response Box (Psychology Software Tools, Schneider, (1995). The accuracy of the response was manually collected by the researcher using a keyboard.

#### Procedure

In Experiment 3A and 3B, the task was identical to Experiment 1, except for the instructions and response type. In Experiment 3A, participants were required to perform a direction discrimination task by verbally reporting the direction the stimulus was pointing at (i.e., by saying aloud either “Izquierda” -left- or “Derecha” -right-). In Experiment 3B, participants were asked to perform a colour-discrimination task by verbally reporting the colour of the target. In both experiments, the researcher was in the room with the participant to categorize the response given by the participants.

#### Design

The design was the same as in Experiment 1. Although participants were not randomly assigned to the two experiments, and therefore the explicit and implicit conditions were run as different experiments rather than as between-participants experimental conditions, for the sake of similarity with the analyses performed in Experiment 1, we performed a 3-factor mixed ANOVA, with Task (explicit vs. implicit) as a between-participants factor, and Target Type and Congruency as within-participants factors. Then, a different Target Type X Congruency ANOVA was conducted on the data from each experiment, Experiment 3A: Explicit Task and Experiment 3B: Implicit Task.

### Results

Trials in which the microphone did not correctly record participants’ responses (2.13% for Exp. 3A, and 3.72% for Exp. 3B), trials with an incorrect response (2.12% for Exp. 3A, and 1.15% for Exp. 3B) and correct response trials with RTs below 200 ms (0.03% for Exp. 3A, and 0.00% for Exp. 3B) or above 1300 ms (0.95% for Exp. 3A, and 0.31% for Exp. 3B) were excluded from the RT analysis. Mean (and standard deviation) for RTs and percentages of errors (% IR) for each experimental condition are shown in Table 3.

**Table 3:** Mean correct RTs (in ms) and percentage of incorrect responses (IR) (with the corresponding standard deviations—SD—, in parentheses) for each experimental condition in Explicit Verbal Task (Exp. 3A) and Implicit Verbal Task (Exp. 3B)

		Arrow				Eye-Gaze			
		RT	SD	%IR	SD	RT	SD	%IR	SD
Explicit Verbal Task	Congruent	765	85	1.33	1.87	839	90	2.12	1.83
	Incongruent	782	85	1.94	2.47	825	76	3.04	2.50
Implicit Verbal Task	Congruent	649	79	1.19	1.43	669	60	1.25	1.59
	Incongruent	645	76	1.24	1.44	668	60	0.96	1.22

As in Experiment 1, the analysis revealed a main effect of Target Type, *F*(1,48) = 61.80, MSE = 1300, *p* < 0.001, *η*^*2*^*p* = 0.56, and a main effect of Task, *F*(1,48) = 47.68, MSE = 22,069, *p* < 0.001, *η*^*2*^*p* = 0.50. The critical three-way interaction between Target Type, Congruency, and Task was significant, *F*(1, 48) = 18.30, MSE = 187, *p* < 0.001, *η*^2^*p* = 0.28, suggesting that the Target Type × Congruency interaction was modulated by the Type of Task. Then a different Target Type × Congruency ANOVA was conducted on the data from each experiment (explicit and implicit tasks).

## Experiment 3A: Explicit Verbal Task

**Fig. 4:**
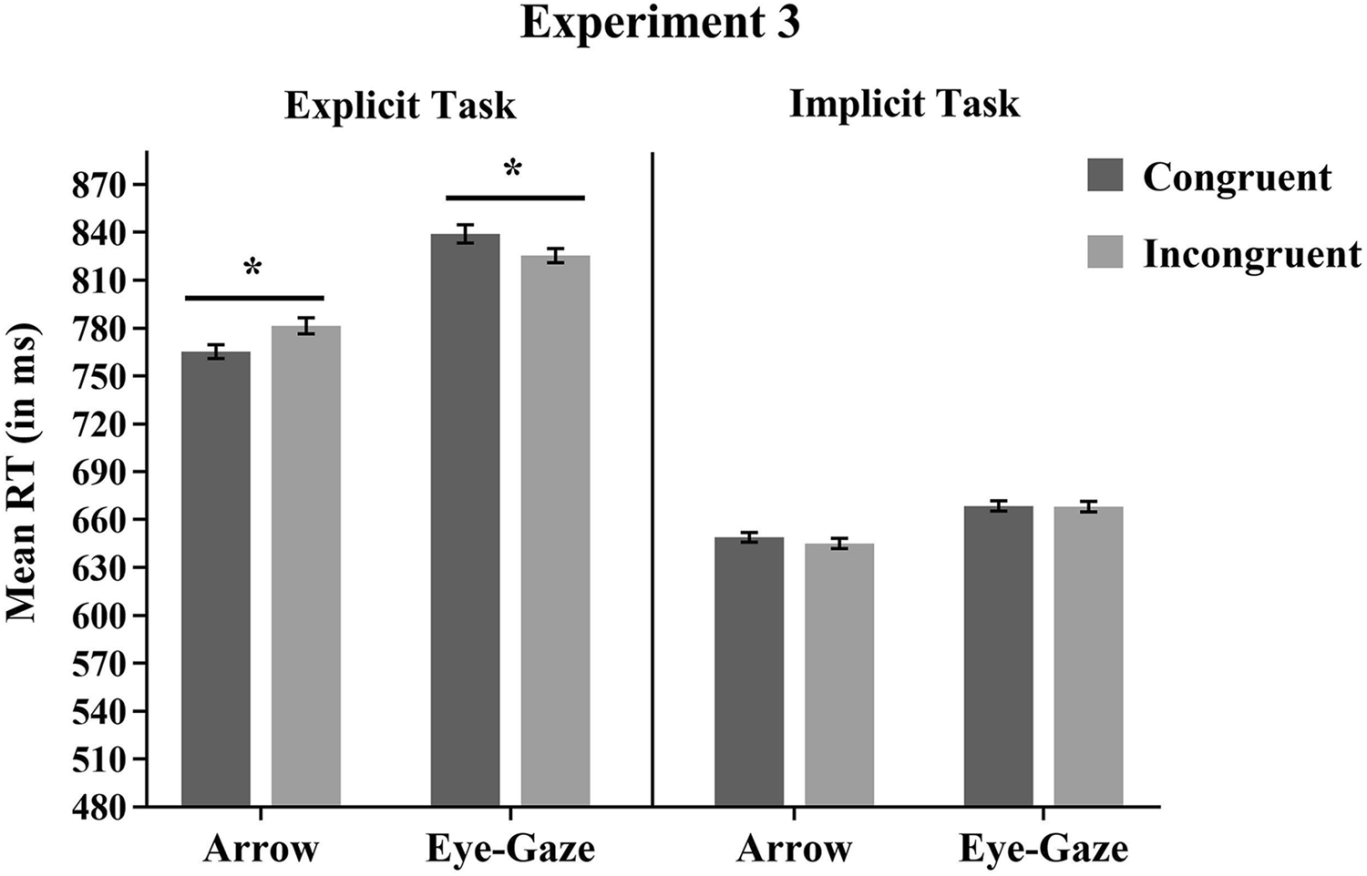
Mean RT for each Target Type and Congruency condition for Experiment 3A (Explicit Verbal Task) and 3B (Implicit Verbal Task). Asterisks represent statistically significant planned comparisons. Cousineau’s method (2005) was used to calculate the standard errors of the means represented as error bars

The analysis revealed a main effect of Target Type, *F*(1,24) = 48.48, MSE = 1785, *p* < 0.001, *η*^*2*^*p* = 0.67, showing faster RTs for arrow targets (773 ms) compared to eye gaze targets (832 ms). The main effect of Congruency was not significant, *F* < 1. The Target Type × Congruency interaction was significant (*F*(1,24) = 20.72, MSE = 269, *p* < 0.001, *η*^*2*^*p* = 0.46). Planned comparisons showed that with arrow stimuli, RTs were significantly slower for incongruent (782 ms) than for congruent trials (765 ms) *F*(1,24) = 15.60, MSE = 213, *p* < 0.001, *η*^*2*^*p* = 0.39. In contrast, for eye-gaze stimuli, RTs were significantly faster for incongruent (825 ms) than for congruent trials (839 ms), *F*(1,24) = 5.55, MSE = 415, *p* = 0.027, *η*^*2*^*p* = 0.19 (see Fig. 4).

The analysis of errors showed a main effect of Target Type, *F*(1,24) = 8.73, MSE = 3, *p* = 0.007 *η*^*2*^*p* = 0.27, with more errors for eye-gaze (2.58%) than arrow trials (1.63%). The main effect of Congruency was significant, *F*(1,24) = 4.75, MSE = 3, *p* = 0.039 *η*^*2*^*p* = 0.17, with more errors for incongruent (2.49%) than congruent trials (1.72%). The interaction between Target Type and Congruency was not significant, *F* < 1. Planned comparisons revealed no significant differences between congruent and incongruent trials neither with arrow stimuli nor with eye-gaze stimuli (ps > 0.11).

## Experiment 3B: Implicit Verbal Task

The analysis also revealed a main effect of Target Type, *F*(1,24) = 13.96, MSE = 814, *p* = 0.001, *η*^*2*^*p* = 0.37, showing faster RTs for arrow targets (647 ms) compared to eye gaze targets (668 ms). Neither the main effect of Congruency nor the interaction between Target Type and Congruency were significant (*F*(1,24) = 1.01, MSE = 104, *p* = 0.325, *η*^*2*^*p* = 0.04 and *F* < 1, respectively). Planned comparisons revealed no significant differences between congruent and incongruent trials neither for arrow nor for eye gaze stimuli (both ps > 0.17) (see Fig. 4).

In the analysis of errors, neither the main effects nor the interaction were significant (all ps > 0.53).

### Discussion

Experiment 3 replicated all the crucial findings of Experiment 1. In particular, when participants were required to verbally respond to the direction of the stimuli (Experiment 3A), standard congruency effects and RCE were observed for arrows and eye-gaze stimuli, respectively. On the other hand, when participants were required to verbally report the colour of the stimuli (Experiment 3B), no congruency effects were observed with either arrow or eye-gaze stimuli, as in the implicit task of Experiments 1 and 2. Since, in the present experiment, responses were verbal instead of manual, these findings suggest that the dissociation between the explicit and implicit task observed in Experiment 1 was due to differences in the way target direction is processed in explicit and implicit tasks rather than to the different manual motor components implicated in the tasks. In order words, differences in spatial congruency effects between explicit and implicit tasks are still observed even in the absence of a lateralized motor response.

Interestingly, although the findings of Experiment 1 were replicated in Experiment 3, overall RT was slower for Experiment 3 than Experiment 1. This suggests that the response modality employed, in this case, verbal response, affects overall RTs in different ways. It has been demonstrated that verbal response interference excess manual response interference (Augustinova et al., 2019; MacLeod, 1991). Furthermore, in Experiment 3, RT was slower for the explicit than the implicit task in contrast with Experiment 1, in which RT was faster for the explicit than the implicit task. Perhaps the lateralized manual response helped performance in the explicit task, when participants were to respond with a natural mapping to the direction of stimuli, but not when they had to arbitrarily categorize colours with left and right responses. In contrast, naming colour might be more natural than naming left–right lateralized responses, thus explaining the main effect of the task in Experiment 3. In any case, given that the RCE observed with gaze was not affected by the manual vs. verbal response, results reinforce the idea that this specific effect of gaze is not related to response factors. Furthermore, results suggest that the fact that congruency effects are only observed with the explicit task cannot be associated with any possible slowdown in RT in the implicit task as observed in Experiments 1 and 2.

## General discussion

In the reported experiments, we examined whether the implicit or incidental processing of gaze and arrows direction can modulate spatial interference effects in general, and the RCE observed with gaze in particular. To this aim, explicit and implicit variants of a spatial interference task were used. In the explicit variant, the direction of the stimuli (eye-gaze or arrows), either congruent or incongruent with their spatial position, was the task-relevant dimension. In the implicit variant, the task-relevant dimension was their colour (blue or brown), a spatially irrelevant dimension.

We observed that responses were, in general, slower for gaze than for arrow stimuli (Hietanen et al., 2006; Vlamings et al., 2005). This may have been due to the social meaning and perceptual complexity of eye-gaze stimuli, which may produce larger attentional capture (Marotta et al., 2018). Supporting the idea that eye-gaze stimuli are special because of their social meaning, Cañadas and Lupiáñez (2012) showed that when triangles or symbolic eyes were used as stimuli, no differences between eye gaze and arrows were found.

Nevertheless, the most important result was the dissociation between the congruency effects observed for arrows and gaze with explicit vs. implicit tasks. When participants were required to respond to the direction of the target stimuli (explicit task), arrows elicited the typical spatial interference effect (i.e., faster RTs when their position was congruent with the direction the arrow was pointing at). According to Luo and Proctor (2013), in the classical Stroop effect or spatial interference effect, the location of stimuli (irrelevant dimension) interferes with their directionality (relevant dimension) creating the spatial congruency effect. In contrast, eye-gaze stimuli produced a RCE (i.e., faster responses for incongruent as compared to congruent trials). This replicates previous findings (Cañadas & Lupiáñez, 2012; Jones, 2015; Marotta et al., 2018, 2019; Torres-Marín et al., 2017) and supports the view according to which attention to eye-gaze may represent a unique attentional process and reflect the operation of a specialized cognitive mechanism (Farroni et al., 2002). On the other hand, when participants were required to respond to the colour of stimuli (implicit task), no congruency effects were observed with either arrows or eye-gaze.

One possible explanation about the lack of congruency effect in the implicit variant of the task could be related to the type of spatial interference paradigm. When participants are required to discriminate another dimension of the stimulus (i.e., the colour), different from the spatial dimension, the congruency effects associated with the spatial interference task could be unaffected, since space is task-irrelevant. Luo and Proctor (2013) proposed that in interference tasks (such as Stroop and Simon), the irrelevant stimulus dimension disturbs performance only when it overlaps with the relevant stimulus dimension. In the implicit task used in the present research, there was a non-spatial dimension (colour) and two spatial dimensions (spatial location and direction of stimuli). The non-spatial dimension and the two spatial dimensions are assumed to be processed by separate systems, each of which operates on its own codes. Therefore, it is possible that the interference effect completely disappeared for both types of stimuli (arrow and eye-gaze) because of the lack of dimensions overlap (Luo & Proctor, 2013; Pang et al., 2020).

Another interpretation is also possible: although the arrow/gaze direction and location dimensions are irrelevant to the task, they could interfere with the lateralized response in 50% of the trials. Therefore, participants might try to inhibit the response to avoid interference. This could have eliminated spatial interference in the implicit task of Experiments 1 and 2. However, this interpretation cannot explain the pattern of results observed in Experiment 3, where responses were not lateralized, and therefore arrow/gaze direction or location should not interfere with responses. Furthermore, we observed typical Simon and Response Mapping compatibility effects for both the arrow and gaze stimuli, which indicates that spatial dimensions could not be completely inhibited. Interestingly, a tendency for a spatial congruency effect was observed in the implicit task when we only considered trials mirroring the compatible mapping of the explicit task. Therefore, the compatible mapping might have incidentally drawn attention to the direction of the stimuli (always in the explicit task, and on the consecutively compatible trials in the implicit one). Thus, some attention to the direction dimension seems to be necessary for the spatial congruency effect to occurs, no matter whether attention is drawn explicitly or implicitly.

It could be argued that with a more elaborated and more ecological social context, like when presenting the whole faces as in Cañadas and Lupiáñez (2012), the RCE would have been observed even at an implicit level. However, this is not a simple issue. Apart from whole faces being more ecological than cropped eyes, the RCE has been observed to be larger with whole faces than with eyes in explicit tasks, where the direction is relevant to the response (Cañadas & Lupiáñez, 2012). However, this might be for a different reason. As shown by Román-Caballero et al., (2021a, 2021b), this could be due to the fact that the Simon effect, which is present for both arrows and gaze, is eliminated when the targets (either arrows or eyes) are surrounded by a complex background, i.e., the whole face, from which they need to be segregated. The cropped eye stimuli are the same from the whole face, and therefore we consider that the same result would be observed with whole faces (i.e., no implicit RCE) as similar effects are observed for cropped eyes and whole faces when the effect of the background is taken into account (see Román-Caballero et al., 2021b).

In any case, the implicit task used in this study was a tool to test the eye-contact hypothesis, since the same eye-contact should still occur on incongruent trials even if participants are responding to a non-spatial dimension. Indeed, previous studies have observed that eye-contact also occurs when gaze is incidentally processed (Adolphs, 2009; Lieberman, 2007; Sato et al., 2016; Stein et al., 2011; Xu et al., 2011). Moreover, the eye-contact effect is observed in detection tasks (Song et al., 2021; lateralized faces or eyes) and in colour-discrimination tasks (see Hietanen et al., 2016) when gaze direction is completely irrelevant to the task. However, our results clearly show that the RCE is not observed when participants do not respond to gaze direction (in spite of paying attention to the eyes, and using the same parameters and stimuli than when a clear RCE is observed in the explicit task). We can therefore conclude that either: (a) No eye-contact occurs, when gaze direction is irrelevant, in the exact stimuli conditions in which a RCE occurs when gaze direction is relevant, or (b) eye contact does occur but it does not affect responses as to produce the RCE. In any case, no matter whether either a) or b) is true, the eye-contact hypothesis of the RCE proposed by Cañadas and Lupiáñez (2012) and Marotta et al. (2018) is refuted.

Moreover, the dissociation in the congruency effect in the explicit and implicit tasks was observed with both manual (Experiment 1 and 2) and verbal (Experiment 3) responses. We hypothesized that response mapping might have influenced the outcome of the study and, thus, our understanding of the results. For example, the difference in congruency effects observed between explicit and implicit tasks might be related to the different mapping of responses. Indeed, while in the explicit task, there was always a compatible mapping between the target direction and the response location (e.g., an eye-gaze/arrow indicating left always required a left response), in the implicit task, response mapping was arbitrary. Therefore, a compatible response mapping was only required for 50% of trials. However, the combined analysis of Experiment 1 and 2 considering response mapping as a factor showed that the effect of response mapping was the same independently of the type of the stimuli (arrow or eye-gaze) used and therefore it was not able to explain the RCE observed for eye-gaze stimuli. In addition, the fact that the same dissociation between implicit and explicit tasks was observed with both manual and verbal responses suggests that this dissociation is due to actual differences in the way target direction is processed in explicit vs. implicit tasks rather than to different manual motor components implicated in these two tasks. Therefore, all explanations of the eye-gaze RCE that lay on response lateralization factors are refuted by this finding.

An alternative explanation to the eye-contact hypothesis might be that the RCE observed with eye-gaze stimuli is related to the fact that the gaze can lead to an approach or avoidance of social behaviour. In incongruent trials, gaze direction may be interpreted as a social approach, while in congruent trials, it may be interpreted as a situation of social distancing or avoidance. This may respectively lead to more or less engagement to the task (Hietanen, 2018; Hietanen et al., 2008). However, the results of the implicit task do not seem to support this explanation. Indeed, when participants had to discriminate another characteristic of the target, although eye-contact or approach behaviour is maintained, no RCE was observed.

Yet another different explanation is related to the joint attention behaviour (Cañadas & Lupiáñez, 2012; Edwards et al., 2020; Mundy, 2018). It could be argued that participants and the eye-gaze stimulus share the same focus of attention (i.e., fixation point) when an incongruent trial is presented (like in the example of Fig. 1), consequently facilitating the processing of the gaze. In congruent trials, however, eye-gaze is averted away from the central point of mutual attention, looking at out of the task. Therefore, in congruent trials, it is possible that our attention and eyes are moved outside the task, causing what might be called “joint distraction”, i.e., drawing attention outside of the task and therefore increasing RTs (see Hemmerich et al., 2021, for an extended explanation of this hypothesis).

The three explanations described above explain the RCE (eye-contact, approach/avoidance, and joint attention/joint distraction) share characteristics related to the social properties of eye-gaze stimuli. Although there are indeed some commonalities between the three explanations, it is also important to acknowledge that there are different social modulators of eye-gaze stimuli that might lead to different levels of processing of gaze and therefore to different effects on human visual attention that need to be understood and investigated (Dalmaso et al., 2020). According to the literature, eye-contact (Chen & Yeh, 2012; Rothkirch et al., 2015; Stein et al., 2011) and approach-avoidance motivational theory (Elliot, 2006; Reichardt, 2018) can occur even in implicit tasks, as their effect is a rapid and automatic response, which does not require explicit attentional behaviour towards the stimulus to generate a response (Hietanen, 2018). Therefore, if these explanations underlined the RCE, the same results would have occurred in both implicit and explicit tasks. Then, only the joint attention/joint distraction explanation would remain as a potential explanation, as explicit processing of gaze direction seems to be an important condition for generating the RCE. Both joint attention/joint distraction would require developing gaze intention, even if automatically, and the following of gaze in a particular direction, which can happen automatically in explicit tasks.

In the case of gaze cueing tasks, in which participants must respond to a lateralized object, the presentation of a face at fixation benefits processing of gazed-at objects, even if the gaze is non-predictive and completely irrelevant for the task. However, as described above, similar cueing effects are observed for gaze and arrows (Brignani et al., 2009), even if gaze cueing effects have been attributed to joint attention (Kawai, 2011; Xu et al., 2011). The fact that gaze cueing seems to occur with implicit processing of gaze, whereas for the RCE explicit processing of gaze direction is necessary, would indicate that different mechanisms underlie the two effects. The presence of the lateralized object might incidentally activate processing of gaze direction in the gaze cueing paradigm, whereas in the spatial interference paradigm, gaze direction is only intentionally activated when it is task-relevant. Although Edwards et al. (2020) have attributed the RCE to joint attention, i.e., the beneficial effect of both the participant and the gaze to look at the fixation point in incongruent trials, Aranda‐Martín et al. (2022) have recently shown that the RCE does not appear until late childhood, in spite of joint attention being fully developed much earlier (Mundy et al., 2007). This would leave “joint distraction” (Hemmerich et al., 2021) as the more likely explanation of the RCE.

This “joint distraction” or whatever mechanism is producing the RCE, would be exclusive of gaze, in contrast to other orienting mechanisms, also present in gaze, but shared with non-social stimuli like arrows, as observed with gaze cueing paradigms (Bonmassar et al., 2019; Brignani et al., 2009). Therefore, gaze seems to trigger domain-general and domain-specific orienting mechanisms. The results of a recent ERP study from our laboratory with the same spatial interference paradigm seems to support this claim. Indeed, we observed comparable congruency modulations for both eye-gaze and arrows stimuli at early stages of processing (P1, N1, and N170; i.e., domain-general effect) and later dissociations (N2 and P3; i.e., domain-specific effect) according to the type of target (Marotta et al., 2019). This may suggest that the initial attention and perceptual stages of stimuli processing (maybe related to automatic mechanisms) are similar for gaze and arrows, while later stages (maybe related to the controlled aspects of social attention) differ according to the type of the stimulus used (Capozzi & Ristic, 2020).

To sum up, the results of this work can be summarized in some key contributions. First, we replicated the findings in which in the context of a spatial interference task, arrow and eye gaze generate opposite congruency effects, a classical congruency effect with arrows and a RCE with eye-gaze stimuli, when there is an explicit processing of the direction of stimuli. Second, the congruency effect with arrow stimuli and RCE with eye gaze stimuli disappeared in the implicit task, when the direction of stimuli was not relevant for the task. Finally, the congruency effect with the arrow and RCE with eye-gaze appeared in the explicit task even when the response was verbal, demonstrating that it is not necessary to generate a lateralized motor response to observe the above-mention effects.

Beyond the finding of a good explanation for the RCE with eye-gaze stimuli, data from the present research suggests that both eye-gaze and arrow direction significantly affect behaviour in a spatial interference paradigm only when they are task-relevant and explicitly processed. On the other hand, the fact that the RCE with eye-gaze stimuli was absent in the implicit task, both when the response was manual and when it was verbal, is not consistent with the eye-contact hypothesis (Cañadas & Lupiáñez, 2012; Marotta et al., 2018, 2019), since several studies have shown that eye-contact can be processed automatically and involuntarily (Mares et al., 2016; Sato et al., 2007; Stein et al., 2011; Xu et al., 2011). Furthermore, this finding is also relevant to dismiss any explanation based on response compatibility factors. Future research should keep investigating alternative plausible explanations as joint attention or joint distraction effects exclusively elicited by eye-gaze.

## Data Availability

Readers seeking access to the data and experimental materials should contact the author Cristina Narganes-Pineda (cnarganes@ugr.es). Data and experimental materials are also available in the Open Science Framework repository (https://osf.io/aetg7/). No part of the study procedure or analyses were pre-registered prior to the research being conducted.
